# Basophil activation test in Hymenoptera venom allergy 

**DOI:** 10.5414/ALX02522E

**Published:** 2024-08-19

**Authors:** Bernadette Eberlein, Knut Brockow, Ulf Darsow, Tilo Biedermann, Simon Blank

**Affiliations:** 1 Department of Dermatology and Allergy Biederstein, School of Medicine and Health, Technical University of Munich, and; 2 Center of Allergy and Environment (ZAUM), Technical University of Munich, School of Medicine and Helmholtz Center Munich, German Research Center for Environmental Health, Munich, Germany

**Keywords:** basophil activation test, Hymenoptera venom allergy, component resolved diagnosis, venom immunotherapy

## Abstract

Before starting venom-specific immunotherapy (VIT), systemic sting reactions to Hymenoptera venoms require allergological workup in order to prove an IgE-mediated reaction and to identify the culprit insect venom. In addition to skin tests and the determination of specific IgE antibodies, the basophil activation test (BAT) using flow cytometry has emerged as a powerful tool and sensitive marker for this purpose in recent years. BAT seems to have a better informative value in terms of clinical relevance compared to the other tests. In Hymenoptera venom allergies, BAT is particularly useful for the diagnosis of cases with unclear or contradictory history and sensitization profile. Its results are associated with adverse reactions during VIT and efficacy of VIT and therefore have a certain predictive value for side effects and treatment failure of VIT. In research, it is mainly used to characterize the allergenic components of Hymenoptera venoms. This review article focuses on these topics.

## Introduction 

In the United States and Europe, ~ 3% of the general population report systemic sting reactions after Hymenoptera stings. In patients with such a history, an indication for venom immunotherapy (VIT) should be considered. This requires evidence of IgE-mediated Hymenoptera venom sensitization, and the decision as to which venom should be used for VIT is based on history and allergy test results. For the determination of Hymenoptera venom-specific IgE (sIgE) antibodies a stepwise diagnosis using whole venoms and allergen components is recommended. Skin testing with venoms should be performed, if no sIgE is detected or if there is a discrepancy between the history and in vitro findings. In the case of contraindications of skin tests, of double sensitization to bee and vespid venom, or if a false negative result for the causative venom is suspected, cellular tests can be performed [1]. 

Despite many immunological effects induced by VIT, clinical protection is not always achieved, and means to verify VIT-induced tolerance are sought for. Determining the occurrence of a protective effect requires a sting provocation or a field sting by the culprit insect [1]. It would be very helpful, if the clinical efficacy of VIT could be determined based only on laboratory parameters. There is evidence that basophil activation tests (BAT) are helpful additional tests that can predict the severity of adverse reactions and the efficacy of VIT. 

The knowledge of the composition of Hymenoptera venoms and the development of recombinantly produced cross-reactive carbohydrate (CCD)-free Hymenoptera venom allergens has improved the sIgE diagnostics and led to the field of molecular or component-resolved diagnostics (CRD). Identification and characterization of new allergens of Hymenoptera venoms were carried out using biochemical and molecular biological methods including BATs. 

This review article aims to discuss these topics in more detail. 

## Basophil activation test 

In general, cellular in vitro tests can be used for diagnosis of IgE-mediated allergies. In recent years the BAT which measures activation of basophils after incubation with allergens or other triggers by flow cytometry has emerged as the most widely used cellular test for this purpose. 

In most studies, the activation marker CD63 was favored, occasionally also CD203c. CD63, a membrane component of the basophil granules, is not a basophil-specific marker and is also expressed on other blood cells. Therefore, further labeling is necessary for the identification of basophils. Possible markers include CCR3, IgE, CRTH2, CD203c, or CD123. CD203c, an ectoenzyme located both on the plasma membrane and in the cytoplasmic compartment of basophils, is a basophil-specific marker and is expressed constitutively. The test can be performed with full blood, washed basophils, or donor basophils and patient serum (passive BAT). This and various existing protocols are the main differences between the BATs used in different laboratories. The markers CD203c and CD63 are upregulated after IgE receptor aggregation but have partially different metabolic pathways and follow different kinetics. Interleukin-3 potentiates the allergen-induced CD63 expression without upregulating CD63 itself, whereas it increases CD203c expression even without the addition of an allergen. 

Results of the BATs are usually expressed as percentages of activated basophils (% CD63+ cells), sometimes also as MFI (mean fluorescent intensity). This basophil reactivity measures the number of basophils that respond to a given stimulus. Maximal basophil reactivity is the maximal activity induced by a given stimulus. Additionally, further parameters such as the determination of the half-maximal allergen concentration (EC50, CD-sens, basophil sensitivity), the calculation of a ratio (CD63 ratio) of allergen-induced CD63 activation in comparison to an IgE-dependent positive control (anti-IgE or anti-FcεRI) or of the area under the curve (AUC) in dose-response curves turned out to be of value for the assessment of clinically relevant allergies and therapy outcomes [2]. Details can be found in an EAACI position paper [3]. 

## Diagnosis of hymenoptera venom allergy 

For Hymenoptera venom allergies, the sensitivity of the BAT with whole venom extracts varies between 85 and 100% and the specificity between 83 and 100% [2]. There is no correlation between basophil activation and the clinical severity of the previous sting reaction reported by patients [4]. 

BAT can be used as a diagnostic tool for the detection of an IgE-mediated reaction, especially if skin tests and sIgE antibodies to insect venom extracts are negative. Although the component-resolved diagnosis has made significant progress in sIgE determination for Hymenoptera venom-allergic patients, there are still individuals in which only the BAT shows positive diagnostic results [5]. Negative tests including the BAT can confirm the non-IgE mediated reaction after a Hymenoptera sting thus helping in the decision against the performance of VIT. 

The BAT turned out to be helpful also in cases of double sensitization to bee and vespid venom and a clinical reaction to only one insect species or in cases of Hymenoptera stings that cannot be clearly assigned to a particular insect species from the clinical history. In ~ 1/3 of the patients, information about the clinically relevant insect could be obtained by BAT incubating the cells with bee and vespid venom extracts and, if necessary, by calculating the half-maximum concentration of the dose-response curves and forming a ratio [6]. The clinical relevance of such BAT results could be confirmed in patients with double sensitization (skin test and sIgE antibodies) and exclusive monosensitization to vespid venom in the BAT: 93% of the patients tolerated a diagnostic sting challenge test with the bee (BAT negative) without systemic reaction, 7% suffered from a mild systemic reaction [7]. Thus, unnecessary specific immunotherapies can be avoided with the help of BAT. 

Furthermore, it could be shown in 12 double-sensitized patients that BAT presented a higher sensitivity than CAP-inhibition and a positive agreement of 71.4%. Likewise, BAT was able to identify 100% of culprit insects in cases with otherwise inconclusive results [8]. 

The use of recombinantly produced CCD-free Hymenoptera venom allergens led to an improvement of the BAT results compared to the total Hymenoptera venom extracts in part of the patients: 

In 43 patients with vespid venom allergy, the use of the four recombinant allergens rVes v 1, rVes v 2, rVes v 3, and rVes v 5 was investigated using BAT. BAT with rVes v 5 provided a specificity of 100% and a sensitivity of 81%, whereas BAT performed with natural venom showed only a specificity of 94.1% and a sensitivity of 68.3%. Additionally, BAT performed with rVes v 5 followed by rVes v 3 was the most sensitive and specific procedure among all recombinant allergens tested. Also, some patients were detected as being negative for rVes v 5 but positive for other recombinant allergens or conventional venom extract in the BAT. Therefore this test markedly improved the specificity of diagnosis in vespid venom-allergic subjects when compared to respective sIgE detection in serum [9]. 

In honey bee venom-allergic patients, the results were less conclusive. In 8/13 honey bee venom-allergic patients rApi m 10 was able to induce basophil activation upon almost 100% [10]. In 9 patients sensitized to Api m 1 and Api m 2, a conventional BAT with honey bee extract revealed a higher basophil activation compared to the components nApi m 1 and rApi m 2, but in 8 patients sensitized only to Api m 1 the results were comparable [11]. 

In serologically double-sensitized patients, BATs were performed not only with venom extracts but additionally with single components (Api m 1, Api m 10, Ves v 1, and Ves v 5). Results showed that BATs with Ves v 5, followed by Api m 1 and Api m 10, were helpful for the decision for VIT with the clinically relevant insect in 28.6% of these patients [12]. 

## Safety and efficacy of venom immunotherapy 

Several studies showed that BAT can predict the severity of adverse reactions during VIT and the efficacy of VIT: 

In a large study of 322 patients undergoing honey bee VIT, it was shown that BAT response was the best biomarker for severe adverse events during VIT [13]. 

A decrease of basophil activation using mostly submaximal concentrations of insect venom extracts was only observed in part of the studies up to 18 months after the beginning of VIT, whereas a lower basophil reactivity was found throughout all studies after 2 years of treatment, and this effect remained until the completion of a 3- to 5-year immunotherapy period [14, 15, 16]. At the end (mean 4.4 years) of VIT, a significant difference was also shown for submaximal concentrations of bee venom in patients reacting to a sting challenge compared to patients who turned out to be protected [17]. The suppression of the allergen-specific basophil response also lasted 1 year after completing 4 – 6.5 years of immunotherapy [15]. In a BAT inhibition assay incubating blood of donor patients with Hymenoptera venom allergy with sera from patients undergoing VIT for at least 1 year, the basophil response was almost completely inhibited at submaximal allergen concentrations [18]. It was shown that patients who after discontinuation of immunotherapy had systemic allergic reactions to field stings had a persistence of high basophil activation at submaximal concentrations in contrast to protected patients [19]. 

## Characterization of components 

In recent years, identification and characterization of new components of Hymenoptera venoms by biochemical and molecular biological methods have made significant progress, shifting the focus from the whole venom to individual allergenic molecules [20]. Some examples are given at which aspect BAT was helpful in this context: 

Antigens 5 are the most potent allergens in vespid venoms and are found in the venom of nearly all Vespoidea species with a varying degree of sequence homology. BATs were performed in 21 vespid-allergic patients with the recombinantly produced antigens 5 of seven allergy-relevant species: *Vespula vulgaris* (rVes v 5), the hornet *Vespa crabro* (rVesp c 5), the European paper wasp *Polistes dominula* (rPol d 5), the American paper wasp *Polistes annularis* (rPol a 5), the white-faced hornet *Dolichovespula maculata* (Dol m 5), the fire ant *Solenopsis invicta* (Sol i 3), and the wasp *Polybia scutellaris* (rPoly s 5). In BAT, the vespid-allergic patients (mainly sensitized to *Vespula* or *Vespa*) showed different activation profiles in response to the different antigens 5. Six of 20 (30%) patients exhibited basophil activation in response to rVes v 5 and/or rVesp c 5 only. The basophils of further 11 patients (55%) were activated by either all or different combinations of antigens 5. However, in most of these patients, the basophil activation was more pronounced in response to rVes v 5 and/or rVesp c 5. Only in 2 patients, the activation pattern was more distinct in response to other allergens than rVes v 5 and/or rVesp c 5. Also rPoly s 5, which was considered a hypoallergenic antigen 5, was able to activate patient-derived basophils in this assay. These results demonstrated pronounced cross-reactivity of vespid venoms on a molecular basis [21]. 

Another allergen of *Polistes dominula*, the dipeptidyl peptidase IV rPol d 3, showed a basophil activation in *Polistes dominula* venom – and/or *Vespula*-allergic patients from Spain and honey bee- and *Vespula*-allergic patients from Germany and was compared to other recombinant dipeptidyl peptidase IV allergens rVes v 3 or rApi m 5. For most patients BATs with dipeptidyl peptidases IV would have been able to identify the insect(s) to which the patients were most likely primarily sensitized [22]. 

Polistes PLA2 from *Polistes dominula* venom and the honey bee venom components C1q-like protein (C1q) and PDGF/VEGF-like (PVF1) were unable to activate basophils of allergic patients despite exhibiting sIgE reactivity questioning their role in the context of clinically relevant sensitization. Similarly, neither the hyaluronidase of *Polistes dominula* (Pol d 2) nor of *Vespula vulgaris* (Ves v 2.0201) showed significant basophil activation in any Hymenoptera venom-allergic patient, whereas the allergen rApi m 2 caused a moderate activation in Api m 2-sensitized honey bee venom-allergic patients [23, 24]. 

## Limitations 

Around 10 – 15% of the patients are non-responders with basophils neither inducing a CD63 nor CD203c activation to allergen stimulation and to positive controls through anti-IgE and/or -FcεRI. This is attributed to differences in the intracellular signaling pathway of these receptors, particularly in the expression of Syk [3]. In these cases, results are not interpretable. Mast cell activation tests represent an alternative diagnostic tool for these non-responders with Hymenoptera venom allergy [25]. 

In patients with mastocytosis, BAT can be negative despite a clear history of anaphylaxis to Hymenoptera venoms. This might be dependent on the basophil markers used. In cases where CCR3 is used as a basophil marker, basophils with low amounts of IgE on their surface are likely to be selected, explaining the poor results of the BAT. Detecting basophils in BAT with an anti-IgE antibody would clearly improve the results and make it possible to avoid false negatives [26]. 

## Conclusion and perspectives 

This overview showed that the BAT with whole venom extracts and allergen components is able to help in the diagnosis of Hymenoptera venom allergy, especially in double-sensitized patients and patients with negative results in routine diagnostics. 

Furthermore, the results of basophil activation tests correspond to the success and tolerance of VIT using submaximal allergen concentrations. The repeated application of BAT in all patients undergoing VIT is unlikely to be routinely performed due to the effort required for testing. When sting tests are not available but a patient is at particular risk of treatment failure, the BAT could be a piece of the puzzle in the assessment of the patient’s risk of treatment failure. 

For investigations of allergen components, BAT can add important information in terms of cross-reactivity or lack of allergenicity (Table 1). 

Standardization and automatization of this cellular test are expected to expand its use for the above-mentioned indications. 

## Authors’ contributions 

B. Eberlein wrote the manuscript. K. Brockow, U. Darsow, T. Biedermann, and S. Blank revised the manuscript. 

## Funding 

No funding was provided for the preparation of this manuscript. 

## Conflict of interest 

BE reports non-financial support from Bühlmann Laboratories outside the submitted work. KB received lecture fees from ALK, Bencard and ThermoFisher and reports non-financial support from Bühlmann Laboratories outside the submitted work. SB reports grants and personal fees from Bencard, Thermo Fisher Scientific and Allergy Therapeutics, grants from Leti Pharma, and non-financial support from Siemens Healthineers, outside the submitted work. The rest of the authors declare that they have no relevant conflicts of interest. 

**Table 1.** Overview of possible current applications of basophil activation test for Hymenoptera venom allergy.

**Field of application**	**Group of patients**	**Allergens to be used in the BAT**
Diagnosis of Hymenoptera venom allergy	Patients with negative results in routine testing	Whole venom extracts, allergen components
	Patients with double sensitization	Whole venom extracts, allergen components
Efficacy and tolerance of VIT	Patients with adverse events during VIT	Submaximal concentrations of whole venom extracts in the course of VIT
	Patients with systemic reactions after a field sting or sting challenge during VIT	Submaximal concentrations of whole venom extracts in the course of VIT
Characterization of allergen components	Patients with a history or sensitization profile relevant to the scientific issue	Allergen components

BAT = basophil activation test; VIT = venom-specific immunotherapy.

## References

[1] Ruëff F Bauer A Becker S Brehler R Brockow K Chaker AM Darsow U Fischer J Fuchs T Gerstlauer M Gernert S Hamelmann E Hötzenecker W Klimek L Lange L Merk H Mülleneisen NK Neustädter I Pfützner W Sieber W Diagnosis and treatment of Hymenoptera venom allergy: S2k Guideline of the German Society of Allergology and Clinical Immunology (DGAKI) in collaboration with the Arbeitsgemeinschaft für Berufs- und Umweltdermatologie e.V. (ABD), the Medical Association of German Allergologists (AeDA), the German Society of Dermatology (DDG), the German Society of Oto-Rhino-Laryngology, Head and Neck Surgery (DGHNOKC), the German Society of Pediatrics and Adolescent Medicine (DGKJ), the Society for Pediatric Allergy and Environmental Medicine (GPA), German Respiratory Society (DGP), and the Austrian Society for Allergy and Immunology (ÖGAI). Allergol Select. 2023; 7: 154–190. 37854067 10.5414/ALX02430E PMC10580978

[2] Eberlein B Basophil activation as marker of clinically relevant allergy and therapy outcome. Front Immunol. 2020; 11: 1815. 32973757 10.3389/fimmu.2020.01815 PMC7472882

[3] Hoffmann HJ Santos AF Mayorga C Nopp A Eberlein B Ferrer M Rouzaire P Ebo DG Sabato V Sanz ML Pecaric-Petkovic T Patil SU Hausmann OV Shreffler WG Korosec P Knol EF The clinical utility of basophil activation testing in diagnosis and monitoring of allergic disease. Allergy. 2015; 70: 1393–1405. 26198455 10.1111/all.12698

[4] Eberlein-König B Schmidt-Leidescher C Rakoski J Behrendt H Ring J In vitro basophil activation using CD63 expression in patients with bee and wasp venom allergy. J Investig Allergol Clin Immunol. 2006; 16: 5–10. 16599242

[5] Korosec P Erzen R Silar M Bajrovic N Kopac P Kosnik M Basophil responsiveness in patients with insect sting allergies and negative venom-specific immunoglobulin E and skin prick test results. Clin Exp Allergy. 2009; 39: 1730–1737. 19689457 10.1111/j.1365-2222.2009.03347.x

[6] Eberlein B Krischan L Darsow U Ollert M Ring J Double positivity to bee and wasp venom: improved diagnostic procedure by recombinant allergen-based IgE testing and basophil activation test including data about cross-reactive carbohydrate determinants. J Allergy Clin Immunol. 2012; 130: 155–161. 22421265 10.1016/j.jaci.2012.02.008

[7] Bokanovic D Arzt-Gradwohl L Schwarz I Schrautzer C Laipold K Aberer W Binder B Sturm GJ Possible utility of basophil activation test in dual honeybee and vespid sensitization. J Allergy Clin Immunol Pract. 2020; 8: 392–394. 31233939 10.1016/j.jaip.2019.06.008

[8] Cabrera CM Palacios-Cañas A Joyanes-Romo JB Urra JM Mur P Basophil activation test as alternative method to CAP-inhibition in patients with double sensitization to vespid venoms. Mol Immunol. 2022; 149: 59–65. 35749834 10.1016/j.molimm.2022.06.002

[9] Balzer L Pennino D Blank S Seismann H Darsow U Schnedler M McIntyre M Ollert MW Durham SR Spillner E Ring J Cifuentes L Basophil activation test using recombinant allergens: highly specific diagnostic method complementing routine tests in wasp venom allergy. PLoS One. 2014; 9: e108619. 25329342 10.1371/journal.pone.0108619 PMC4201461

[10] Blank S Seismann H Michel Y McIntyre M Cifuentes L Braren I Grunwald T Darsow U Ring J Bredehorst R Ollert M Spillner E Api m 10, a genuine A. mellifera venom allergen, is clinically relevant but underrepresented in therapeutic extracts. Allergy. 2011; 66: 1322–1329. 21658068 10.1111/j.1398-9995.2011.02667.x

[11] Koren A Lunder M Molek P Kopač P Zahirović A Gattinger P Mittermann I Valenta R Korošec P Fluorescent labeling of major honeybee allergens Api m 1 and Api m 2 with quantum dots and the development of a multiplex basophil activation test. Allergy. 2020; 75: 1753–1756. 31950504 10.1111/all.14185

[12] Schmidle P Blank S Altrichter S Hoetzenecker W Brockow K Darsow U Biedermann T Eberlein B Basophil activation test in double-sensitized patients with hymenoptera venom allergy: Additional benefit of component-resolved diagnostics. J Allergy Clin Immunol Pract. 2023; 11: 2890–2899. 37302791 10.1016/j.jaip.2023.06.007

[13] Kopač P Custovic A Zidarn M Šilar M Šelb J Bajrović N Eržen R Košnik M Korošec P Biomarkers of the severity of honeybee sting reactions and the severity and threshold of systemic adverse events during immunotherapy. J Allergy Clin Immunol Pract. 2021; 9: 3157–3163. 33962066 10.1016/j.jaip.2021.04.045

[14] Ebo DG Hagendorens MM Schuerwegh AJ Beirens LM Bridts CH De Clerck LS Stevens WJ Flow-assisted quantification of in vitro activated basophils in the diagnosis of wasp venom allergy and follow-up of wasp venom immunotherapy. Cytometry B Clin Cytom. 2007; 72: 196–203. 17111386 10.1002/cyto.b.20142

[15] Eržen R Košnik M Silar M Korošec P Basophil response and the induction of a tolerance in venom immunotherapy: a long-term sting challenge study. Allergy. 2012; 67: 822–830. 22469017 10.1111/j.1398-9995.2012.02817.x

[16] Rodríguez Trabado A Cámara Hijón C Ramos Cantariño A Romero-Chala S García-Trujillo JA Fernández Pereira LM M. Short-, intermediate-, and long-term changes in basophil reactivity induced by venom. Allergy Asthma Immunol Res. 2016; 8: 412–420. 27334779 10.4168/aair.2016.8.5.412 PMC4921695

[17] Kucera P Cvackova M Hulikova K Juzova O Pachl J Basophil activation can predict clinical sensitivity in patients after venom immunotherapy. J Investig Allergol Clin Immunol. 2010; 20: 110–116. 20461965

[18] Arzt L Bokanovic D Schrautzer C Laipold K Möbs C Pfützner W Herzog SA Vollmann J Reider N Bohle B Aberer W Sturm GJ Immunological differences between insect venom-allergic patients with and without immunotherapy and asymptomatically sensitized subjects. Allergy. 2018; 73: 1223–1231. 29171032 10.1111/all.13368

[19] Peternelj A Silar M Erzen R Kosnik M Korosec P Basophil sensitivity in patients not responding to venom immunotherapy. Int Arch Allergy Immunol. 2008; 146: 248–254. 18270492 10.1159/000116361

[20] Dramburg S Hilger C Santos AF de Las Vecillas L Aalberse RC Acevedo N Aglas L Altmann F Arruda KL Asero R Ballmer-Weber B Barber D Beyer K Biedermann T Bilo MB Blank S Bosshard PP Breiteneder H Brough HA Bublin M EAACI Molecular Allergology User’s Guide 2.0. Pediatr Allergy Immunol. 2023; 34: e13854. 37186333 10.1111/pai.13854

[21] Schiener M Eberlein B Moreno-Aguilar C Pietsch G Serrano P McIntyre M Schwarze L Russkamp D Biedermann T Spillner E Darsow U Ollert M Schmidt-Weber CB Blank S Application of recombinant antigen 5 allergens from seven allergy-relevant Hymenoptera species in diagnostics. Allergy. 2017; 72: 98–108. 27496543 10.1111/all.13000

[22] Schiener M Hilger C Eberlein B Pascal M Kuehn A Revets D Planchon S Pietsch G Serrano P Moreno-Aguilar C de la Roca F Biedermann T Darsow U Schmidt-Weber CB Ollert M Blank S The high molecular weight dipeptidyl peptidase IV Pol d 3 is a major allergen of Polistes dominula venom. Sci Rep. 2018; 8: 1318. 29358620 10.1038/s41598-018-19666-7 PMC5778000

[23] Grosch J Eberlein B Waldherr S Pascal M San Bartolomé C De La Roca Pinzón F Dittmar M Hilger C Ollert M Biedermann T Darsow U Bilò MB Schmidt-Weber CB Blank S Characterization of new allergens from the venom of the European paper wasp Polistes dominula. Toxins (Basel). 2021; 13: 559. 34437431 10.3390/toxins13080559 PMC8402607

[24] Russkamp D Van Vaerenbergh M Etzold S Eberlein B Darsow U Schiener M De Smet L Absmaier M Biedermann T Spillner E Ollert M Jakob T Schmidt-Weber CB de Graaf DC Blank S Characterization of the honeybee venom proteins C1q-like protein and PVF1 and their allergenic potential. Toxicon. 2018; 150: 198–206. 29842867 10.1016/j.toxicon.2018.05.017

[25] Koren A Dejanovic L Kopac P Erzen R Bajrovic N Zidarn M Korosec P LAD2 mast cell activation test associates with the reaction severity and diagnoses BAT nonresponders in Hymenoptera venom allergy. J Investig Allergol Clin Immunol. 2023; 0:0. 10.18176/jiaci.0969 37937714

[26] Urra JM Pérez-Lucendo I Extremera A Feo-Brito F Alfaya T The method for selecting basophils might be determinant in the basophil activation test in patients with mastocytosis. J Investig Allergol Clin Immunol. 2020; 30: 65–67. 10.18176/jiaci.0442 31530516

